# Effect of Methyl Jasmonate Elicitation on Triterpene Production and Evaluation of Cytotoxic Activity of Mycelial Culture Extracts of *Ganoderma applanatum* (Pers.) Pat.

**DOI:** 10.3390/plants12020294

**Published:** 2023-01-08

**Authors:** Katarzyna Sułkowska-Ziaja, Agnieszka Galanty, Agnieszka Szewczyk, Paweł Paśko, Katarzyna Kała, Anna Apola, Irma Podolak, Bożena Muszyńska

**Affiliations:** 1 Department of Pharmaceutical Botany, Faculty of Pharmacy, Jagiellonian University Medical College, Medyczna 9, 30-688 Kraków, Poland; 2 Department of Pharmacognosy, Faculty of Pharmacy, Jagiellonian University Medical College, Medyczna 9, 30-688 Kraków, Poland; 3 Department of Food Chemistry and Nutrition, Faculty of Pharmacy, Jagiellonian University Medical College, Medyczna 9, 30-688 Kraków, Poland; 4 Department of Inorganic and Analytical Chemistry, Faculty of Pharmacy, Jagiellonian University Medical College, Medyczna 9, 30-688 Kraków, Poland

**Keywords:** anticancer activity, biotechnology of mushrooms, ganoderic acids, *Ganoderma applanatum*, medicinal mushrooms, submerged cultivation

## Abstract

Abiotic elicitation, a well-known strategy in mushroom biotechnology, promotes increased accumulation of secondary metabolites in mycelial cultures. The study aimed the effects of methyl jasmonate (MeJA) on the production of triterpenes in submerged cultures of *Ganoderma applanatum*. Further, the study evaluated the cytotoxic activity of the extract corresponding to the optimal elicitation variant in selected human cancer cell lines as well as the selectivity against normal cells. MeJA was added on days 1, 4, 6, and 8 in the 10-day growth cycle at concentrations of 10, 50, 100, 150, and 200 µM MeJA. The HPLC-DAD was used to analyze the triterpenes. The cytotoxic activity was tested using the MTTFc assay in grouped panels of skin, prostate, and gastrointestinal cancer cells. The results of the quantitative analyses confirmed the stimulating effect of MeJA on the production of ganoderic acid A and ganoderic acid C. The greatest increase in total triterpenes was found on day 6 of the culture cycle compared to the control group—with the concentration of MeJA—150 µM. Compared to the control samples, mycelial culture extract after the most productive elicitation variant showed significant cytotoxic activity against prostate cancer cells and moderate effects on melanoma cells. *Ganoderma applanatum* mycelial cultures can be proposed as a model to study the dynamics of the accumulation of compounds with therapeutic values through abiotic elicitation.

## 1. Introduction

Evidence from traditional far-Eastern medicine and modern scientific research confirms the potential value of mushrooms as pharmaceutical raw materials [1]. *Ganoderma applanatum* (Pers.) Pat. (*Ganodermataceae*, Basidiomycota), is a parasitic and saprotrophic mushroom widely distributed in the northern hemisphere. The fruiting bodies often develop on living trunks and branches and on the dead wood of deciduous trees, such as *Acer* sp., *Fraxinus* sp., *Fagus* sp., *Tilia* sp., *Populus* sp., *Salix* sp., and *Quercus* sp. They cause severe white rot in the wood [2]. There has been extensive research conducted on the chemical composition of the *Ganoderma applanatum* fruiting bodies, which revealed the presence of diverse groups of chemical compounds. These compounds include polysaccharides, sterols, proteins, and fatty acids [3,4,5]. Additionally, tannins, saponins, phenolic compounds, and flavonoids have also been identified [6,7,8]. Over 380 terpenoids (ganoderic/lucidenic acids, meroterpenoids) were isolated from the fruiting bodies [9]. Studies conducted on the biological activity of the extracts and individual compounds isolated from the fruiting bodies of *Ganoderma applanatum* have confirmed their beneficial properties for therapeutic applications. The biological activities of fruiting bodies include antitumor [10], anti-inflammatory, immunomodulating [11], antioxidant, antibacterial [12,13], hepatoprotective [14], hypoglycemic [15], and antifibrotic effects [16]. Numerous studies conducted on fruiting bodies, including their lanostane derivatives, confirm the significant cytotoxic potential of compounds that have a terpenoid structure. Several reports indicate that these compounds inhibited cell proliferation, angiogenesis, invasion, and metastasis, and induced cell cycle arrest and apoptosis in carcinoma cell lines and animal models [17].

Biotechnological processes play an important role in improving the production of these compounds since the isolation of triterpenes from fruiting bodies is an expensive process [18]. In the broad field of biotechnology, the study of higher fungi biotechnology is currently growing rapidly. The overriding task of the research is to make the best possible use of the biochemical potential of the fungi. Conducting mycelial cultures under laboratory conditions, regardless of climatic conditions, helps in obtaining valuable research material [19]. Further, this method can be used to stimulate the production of valuable secondary metabolites through the influence of various factors during the culture cycle. The mycelial cultures can be treated with elicitors, which can result in the production of secondary metabolites. Elicitation is one of the most promising methods used in plant and fungal biotechnology, which induces stress, thereby increasing their productivity [20]. Elicitation is the manipulation of metabolic and biochemical pathways. In plant and fungal physiology, “stress” is defined as biotic or abiotic factors that have the potential to modify the growth, reproduction, and function of an organism [21]. Abiotic elicitors can include physical factors (such as ultraviolet radiation, temperature, and mechanical damage) and chemicals (such as salicylic acid (SA), jasmonic acid (JA), and methyl jasmonate (MeJA)). The knowledge of the precise mechanism of elicitors’ actions is still limited. The induction of secondary metabolism pathways is considered to increase the ability of an organism to survive in a stressful environment. Elicitors can affect the stimulation of genes associated with the reactions of synthesis, and the accumulation of secondary metabolites [22].

Therefore, the aim of this work is to demonstrate the feasibility of enhancing triterpene production in submerged cultures of *Ganoderma applanatum* by adding MeJA as an exogenous elicitor. The effects of MeJA on the production of ganoderic acids A and ganoderic acids C were comparatively evaluated. Further, the study evaluated the cytotoxic activity of the extract corresponding to the optimal elicitation variant in selected human cancer cell lines as well as the selectivity against normal cells. Cytotoxic potential in human cell lines was assessed, with the cells grouped in three panels, representing the skin (melanoma HTB140 and A375, normal skin keratinocytes HaCaT), prostate (cancer DU145 and PC3, normal human prostate epithelial PNT2), and gastrointestinal tract (colon cancer Caco-2 and HT29, liver HepG2). Additionally, the interaction of the cells with the reference chemotherapeutic agent, doxorubicin, was assessed.

## 2. Results and Discussion

The research involved the study of the mycelial culture of *Ganoderma applanatum*. The experimental cultures were maintained as submerged cultivation. These experiments may provide an alternative source of obtaining medical compounds that are therapeutically valuable [9]. The relatively rapid multiplication of mycelium, compared to the growth of fruiting bodies in nature, is an advantage of following this method. The method can further be used to influence the regulation of mycelial metabolism through the addition of growth promoters, metabolic precursors, or elicitors, which increased the efficiency of the synthesis of bioactive compounds. In this study, MeJA, an abiotic elicitor, was added to the mycelial cultures of *Ganoderma applanatum*, at different concentrations and on selected days of the culture growth cycle. MeJA concentrations in the study were chosen based on data on the impact of the increase in MeJA level compounds in other mycelial cultures of wood decay of mushrooms.

### 2.1. Effect of Elicitor Addition on Biomass Growth

**Figure 1:**
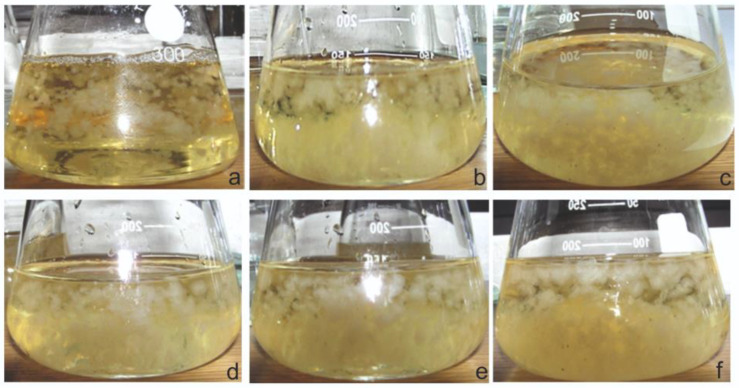
Examples of the morphological appearance of *Ganodema applanatum* control (**a**), and experimental mycelial cultures after 10 days of elicitation. Used concentrations of elicitors: (**b**) 10 µM; (**c**) 50 μM; (**d**) 100 μM; (**e**) 150 μM; (**f**) 200 μM.

The study evaluated the effect of the elicitation on biomass growth and appearance. Throughout the experiment, homogeneous mycelium, free of contamination, was obtained in each culture series. There were also no infections with bacterial or other fungal strains due to regular monitoring by microscopic observations. The result of the analysis proved that the use of MeJA as an elicitor did not inhibit the growth of mycelium biomass (Figure 1).

**Figure 2:**
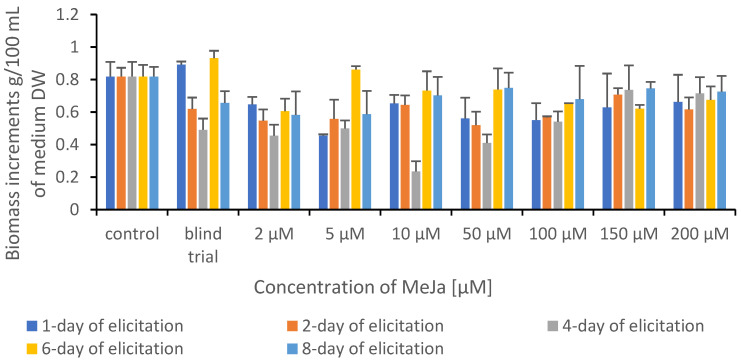
Comparison of biomass increment values of control and experimental cultures of *Ganoderma applanatum* after elicitation.

The biomass increase was assessed by measuring the weight of biomass from each elicitation variant. The biomass was harvested after a 10-day growth cycle, drained from the medium, frozen, and lyophilized. A comparison of biomass increment values of control and experimental cultures after elicitation is shown in Figure 2.

The elicitor addition often caused a decrease in in vitro culture biomass growth while the production of secondary metabolites increased. Such a fact is well known in plant biotechnology studies and has been described before, e.g., MeJA elicitation in *Fagonia indica* adventitious root cultures [23]. In mycelial cultures, the regularity was observed in which the addition of elicitor does not inhibit the growth of mycelium [24].

### 2.2. Effect of Elicitor Addition on Triterpene Content in Mycelial Cultures

Chemical analyses using the HPLC-DAD method confirmed that the addition of the elicitor, MeJA, increased triterpene accumulation in the mycelial cultures of *Ganoderma applanatum*. The addition of MeJA on day 6 of the growth cycle caused the highest increase in triterpene accumulation in the experimental cultures. There was also a significant increase in the content of compounds with the addition of elicitor on day 8 of the culture cycle. The concentration of MeJA added can be a reason for this increase. The addition of MeJA at a concentration of 150 µM on day 6 of the culture cycle resulted in the highest triterpene content, which was a 7.5-fold increase compared to the control sample. On day 8 after MeJA elicitation at a concentration of 150 µM, an increase in total compound content was also observed and was 4.0-fold higher compared to the control sample. It was observed that lower concentrations of MeJA (0–10 µM) did not increase the accumulation of triterpenes in in vitro cultures. The amounts of ganoderic acids A and C in extracts of *Ganoderma applanatum* mycelial cultures after elicitation with methyl jasmonate are shown in Table 1.

The study attempted to increase the accumulation of secondary metabolites with the addition of the elicitor (MeJA), among others, in the mycelial cultures of *Inonotus obliqus*. There was a significant increase in the accumulation of lanostane-type triterpenes when MeJA was used as an elicitor at concentrations of 10–150 µM. The most significant result was a 68.5% increase in accumulation compared to the control sample, using MeJA at a concentration of 50 µM on day 6 of culture [24]. Another study was conducted on the MeJA elicitation of mycelial cultures of *Inonotus baumii*. In this study, the most effective increase in triterpene production was obtained at a MeJA concentration of 150 µM, compared to the control sample. At higher MeJA concentrations (200 and 250 µM), there was an increasingly lower content of triterpene compounds [25]. Additionally, there was an effective elicitation with MeJA in *Ganoderma lucidum*. The content of ganoderic acid was studied before and after the MeJA application. In this study, the optimal concentration of MeJA was 50 µM, where there was a 28.6% increase in the triterpene content compared to the control sample [26].

The studied relationship can be due to the phases of the growth cycle of the mycelial culture. The addition of an elicitor during the logarithmic phase of mycelium growth can result in the optimal effect of the elicitation process. The concentration of the added elicitor is significant: a higher concentration of the elicitor results in a stronger cellular response, which consequently leads to an increased accumulation of secondary metabolites [20]. However, an excessive concentration of the elicitor can have the opposite effect, leading to the activation of defense mechanisms. Consequently, this leads to a decrease in the amount of the accumulated secondary metabolites [22].

In the present study, the identification of the metabolites was performed by HPLC-DAD analysis. The peaks of ganoderic acids A and C were identified by comparing the UV spectra and retention times of the standard compounds. Qualitative analyses were supplemented using the standard internal method. The chemical compounds were quantified against their standards using the calibration curve method.

There are only limited data on the identification of individual compounds from *Ganoderma applanatum* fruit bodies. The first triterpenes isolated from the genus *Ganoderma* were ganoderic acids A and B from *Ganoderma lucidum*; this isolation was made in 1982 by T. Kubota [27].

In turn, the identity of ganoderic acid A and C in *Ganoderma applanatum* was confirmed in 2010 by selective liquid chromatography with tandem mass spectrometry (LC–MS/MS). Mass spectrometry detection was achieved with a triple quadrupole mass spectrometer equipped with an atmospheric pressure chemical ionization interface (APCI) operating in negative and positive ionization modes by switching the polarity once during the run. Due to this method, for the first time, the level of ganoderic acids was accurately determined in the fruiting bodies of *Ganoderma applanatum* [28].

**Figure 3:**
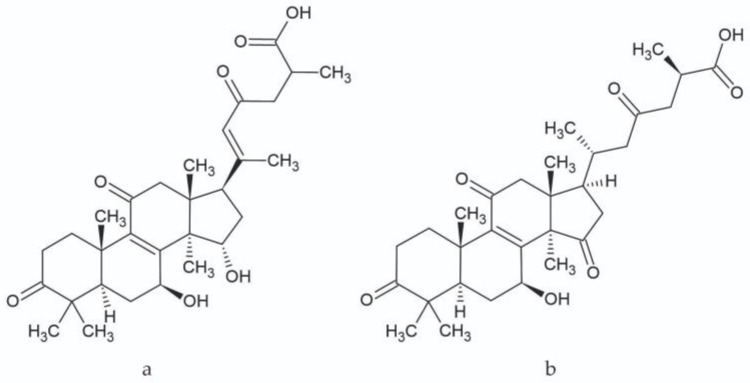
Chemical structure of ganoderic acid A (**a**) and ganoderic acid C (**b**).

The triterpenes present in the studied extracts, which belong to the lanostane type, have multidirectional biological activity along with therapeutic effects [29,30]. In in vitro studies, the activity of ganoderic acid A (Figure 3), among others, was demonstrated along with its antioxidant and anti-inflammatory properties [31]. Additionally, several studies have confirmed the cytotoxic activity in cancer cells—breast cancer, osteosarcoma, or liver cancer [29]. Further studies on the activity of ganoderic acid A can provide multiple opportunities for new applications of this molecule, such as in the treatment of obesity and insulin resistance. The inhibition of the SREBP (Sterol Regulatory Element-Binding Proteins) pathway—a pathway for the biosynthesis of cholesterol, fatty acids, and triglycerides—results in this activity. This compound may prove to be an important structure during the development of drugs to prevent obesity and build insulin resistance [32]. Ganoderic acid C (Figure 3), another compound determined in the research, also has numerous healing properties, such as antitumor, antihistamine, antiaging, and cytotoxic effects. Ganoderic acid C exhibits high inhibitory activity against rat lens aldose reductase (RLAR) with an IC_50_ value of 3.8 µM [33,34].

### 2.3. Validation

The validation parameters presented in Table 2 indicate that the developed method has a satisfactory sensitivity; the limit of detection (LOD) for ganoderic acid A was 0.047 mg/mL, and for ganoderic acid C was 0.068 mg/mL. The limit of quantification (LOQ) was 0.143 and 0.205 mg/mL, respectively. The recovery of the proposed method ranged from 98.57 to 101.19% and demonstrated an adequate accuracy of the method. The analysis of the values of coefficients of variation obtained for the quantitative analyses ranged from 0.15 to 1.44%, indicating suitable precision of the used method. The linearity for the determined substances was maintained in the concentration range of 0.125–1.000 mg/mL.

### 2.4. Cytotoxicity

The chemical analysis results led to the selection of the most productive elicitor testing strategy. The study selected the variable with MeJA added at a concentration of 150 µM on the 6th day of the culture cycle. The extract was compared with the control sample and ganoderic acid A, a reference substance. The selected extracts were screened for cytotoxicity against cancer cells, which were grouped into specially designed panels, i.e., skin, prostate, and gastrointestinal. This in vitro screening model included cancer cells from different malignancies and the corresponding normal cells. This experiment verified the cytotoxic potential of the tested compounds as well as their selectivity. The drug doxorubicin was used as a reference in the study.

The examined extracts and ganoderic acid A revealed varied cytotoxic activity in the tested cancer cell lines. The predominance of E1 (control extract) over E2 (extract obtained after elicitation (variant corresponding to the highest concentration of triterpenes)) was observed within the prostate and skin panel, while E2 extract was more potent to colon cancer cells. In some cases, the activity of the extracts was high (IC_50_ < 20 µg/mL), which was especially observed for E2 extract to Caco2 cells, and E1 extract to DU145 and A375 cells (Table 3).

**Figure 4:**
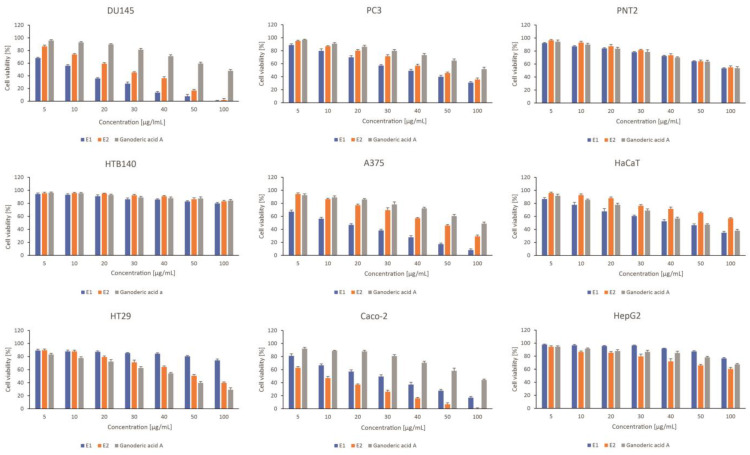
The effect of *Ganoderma applanatum* extracts on viability of prostate cell line panel—DU145, PC3, and PNT2; skin cell line panel—A375, HTB140, HaCaT, and gastrointestinal tract cell lines panel—Caco-2, HT29, HepG2. Cells were treated with E1—control or extract; E2– extract obtained after elicitation (variant corresponding to the highest concentration of triterpenes) and standard substances: ganoderic acid A (*n* = 3). Values are presented as the mean ± SD (standard deviation). Significant differences are shown in Tables S1–S3 (Supplementary Materials).

In general, the extracts were more active in cancer cells characterized by lower malignancies, namely DU145, A375, and Caco2, when compared to the appropriate cancer cells of the same origin but more metastatic (PC3, HTB140, HT29). However, it should be underlined that highly metastatic prostate cancer PC3 cells were affected more strongly by both examined extracts than by doxorubicin, used as a reference cytostatic. Interestingly, ganoderic acid was much less potent to the examined cancer cells, in comparison to the extracts, except for HT29 cells (Figure 4). As far as the toxicity of the tested extracts and ganoderic acid to normal cells used in the study is concerned, they were characterized by high selectivity to prostate PNT2 and liver HepG2 cells. Skin keratinocytes, HaCaT, were moderately affected by E1 extract and ganoderic acid A, but not E2 extract (Figure 4).

Triterpenes are responsible for the cytotoxic activity of the extracts obtained from the fruiting bodies of the genus *Ganoderma*. This activity was determined for lucidadiol, 15α, 26-dihydroxy-5α-lanosta-7,9,24(E)-trien-3-one, and ganoderiol F, and so on. These compounds showed a strong cytotoxic potential against HeLa cells from human cervical cancer, with IC_50_ values of 1, 5, and 8 µM/L, respectively [35].

Other studies have confirmed that triterpene ganoderic acid S inhibits the cell cycle at the S1 phase of HeLa cells, while ganoderic acid Mf inhibits the G1 phase of the HeLa cervical cell line [36]. Further, it has been confirmed that ganoderic acid T can be a natural apoptosis inducer for highly metastatic lung tumors and can also be used to treat other tumor cell lines [37]. The study also attempted to elucidate the mechanism of the induced apoptosis in SGC-7901 cell lines against the *Ganoderma applanatum* extract. The main components determined from this extract were ganoderic acid A and ganoderic acid A [38]. Cytotoxicity against a human colon cancer cell line (Caco-2) was evaluated for the 80% methanol extract, which showed an IC_50_ value of 160 ± 4.08 μg/mL [17].

Several studies conducted in 2022 examined the cytotoxic activity of various species of fungi of the genus *Ganoderma*, including *Ganoderma applanatum*.

Among other things, the cytotoxic effects of extracts obtained from mycelial cultures of six *Ganoderma* species were tested against 16F10 mouse melanoma cells. *Ganoderma carnosum* extract was confirmed to have the highest activity of 43.29% at 0.15 mg/mL [39].

In the same year, a new triterpene was isolated from the ethanolic extract of the fruiting bodies of *Ganoderma lucidum*. This compound exerted moderate antitumor effects (IC_50_ values of 15.38 ± 0.34 and 18.61 ± 0.55 μM against A549 and HepG2, respectively) compared to cisplatin [40]. In addition, both new and previously known compounds with nor-triterpenoid structures were isolated from the fruiting bodies of *Ganoderma lucidum*. The cytotoxic effects of these compounds against LOVO, MCF-7, and RAW264.7 cells were evaluated. Moderate cytotoxicity of the compounds on the mentioned cell lines was demonstrated [41]. The *Ganoderma australe* species also proved to be a rich source of lostane-type triterpenoids: ganodaustralic acids A–G. For ganodaustralic acid G, weak cytotoxicity was demonstrated against SGC-7901 cells [42].

Valuable results were provided by the study of the anticancer potential of *Ganoderma lucidum* against prostate cancer cells (PC-3) by altering the JAK-1/STAT-3 signaling pathway. The findings demonstrate that *Ganoderma lucidum* is convincing for cytotoxicity, ROS accretion, and stimulation of apoptosis in PC-3 cells. Additionally, signal transducer and activating transcription (STAT-3) is a successive oncogenic transcriptional factor that regularizes multiplication and apoptosis in cells. The deletion of STAT-3 transcription was considered an original approach to hinder prostate cell growth [43].

This study extended the existing data on the cytotoxic potential of *Ganoderma applantum*. Additionally, it also provided results on the mycelial cultures of this species for the first time.

## 3. Materials and Methods

### 3.1. Experimental Mycelial Cultures

The initial cultures were established and maintained as reported previously [44]. This study involved the cultivation of mycelium in 300-mililitter Erlenmeyer flasks containing 100 mL of medium. The study used a medium according to Oddoux [45], the composition of which was modified during the many years of research carried out at the Department of Pharmaceutical Botany of the Jagiellonian University Medical College. Modification of the medium composition involved differences in the concentrations of the added macro- and micronutrients. The inoculum used in the study consisted of 20 mL of biomass derived from liquid cultures. Cultures were grown on a rotary shaker (Altel, Kraków, Poland) at 140 rpm. at a temperature of 22 ± 2 °C. All experimental variants included three series of cultures. The voucher specimens of the fruiting bodies and mycelial cultures were deposited in the Department of Pharmaceutical Botany of the Jagiellonian University Medical College.

### 3.2. Elicitation Procedure

The MeJA ethanolic solution was added to the liquid cultures on different days of the growth of the culture. MeJA solutions with the following concentrations were prepared for this purpose in volumetric flasks: 0 (addition of an ethanol solution without MeJA), 2, 5, 10, 50, 100, 150, and 200 µM. A control sample was also examined, to which neither MeJA nor ethanol was added. Each culture was run for 10 days, and MeJA was added on days 1, 2, 4, 6, and 8 of culture growth at a rate of 0.4 mL/flask. After 10 days of culture growth, the resulting biomass was harvested, filtrated from the medium, rinsed with redistilled water, frozen, and lyophilized (freeze dryer, Labconco Corporation, Kansas City, MO, USA). The MeJA solution was sterilized prior to the addition of culture by filtering through a sterile filter (Millipore Milles-Gs 0.22 µm).

### 3.3. Extraction and HPLC Analysis of Triterpenes

Mushroom material harvested from the tested mycelial cultures was frozen and lyophilized (freeze dryer, Labconco Corporation, Kansas City, MO, USA).

The dry biomass was pulverized. Samples (1 g) were extracted using 6 mL of isopropanol (STANLAB, Lublin, Poland) under sonication for 20 min (POLSONIC 2, Warsaw, Poland). Then, the samples were centrifuged (7 min, 2000× *g*; MPW-223E centrifuge; MPW, Warsaw, Poland). The isopropanol supernatants were dried and dissolved in 1 mL of methanol, and then filtered using a 0.22-mictometer Millipore filter (0.22 μm syringe filters; Millex^®^GP; Merck Millipore, Burlington, MA, USA). The samples were analyzed using the HPLC system (Merck-Hitachi, Merck KGaA, Darmstadt, Germany) at λ = 206 nm [46]. A reverse-phase Purospher RP-18e analytical column (4 × 250 nm, 5 mL; Merck) was used with nonlinear gradient elution (90:10, 0 min; 97:3, 10 min) with acetonitrile:water (*v*/*v*) used as mobile phase at a flow rate of 1.0 mL/min. The injection loop was 20 µL, and the temperature of the column was 30 °C. The peaks were identified by comparing UV spectra and retention times of the standard compounds. The qualitative analyses were supplemented using the standard internal method. The chemical compounds were quantified against their standards with the calibration curve method. Chemical standards were purchased from Sigma–Aldrich (Darmstadt, Germany) and MCE–MedChemExpress LLC (New York City, United States). Solvents for the HPLC analysis were purchased from Merck (Darmstadt, Germany).

### 3.4. Validation

Validation of the HPLC-DAD method was performed by determining accuracy, precision, linearity, and LOD and LOQ (European Medicines Agency 2005). Accuracy was determined based on sample analysis of known concentrations and a comparison of the results obtained by a validated method with true values, followed by the calculation of the recovery percentage. Determinations were performed at three concentrations—80, 100, and 120%, and three repetitions were carried out for each level. The precision of the method was determined at three levels of substance concentrations in reference solutions—50, 100, and 150%, and three repetitions were carried out for each level. Linearity was determined by comparing the relationship between the peak area and concentration of tested substances (mg/mL). Two series of assays were made for ganoderic acid A, and ganoderic acid C in the following concentration range from 0.125 to 1.000 mg/mL. LOD and LOQ were determined from the linearity in the concentration range from 0.125 to 1.000 mg/mL for all the tested substances using the following formulas: LOD = 3.3 × Sy/a and LOQ = 10 × Sy/a, where Sy is the estimation error and a is the slope.

### 3.5. Cell Viability Assay

The experiment was conducted on several human cell lines, grouped in three panels, representing skin (melanoma HTB140 and A375, normal skin keratinocytes HaCaT), prostate (cancer DU145 and PC3, normal human prostate epithelial PNT2), and gastrointestinal tract (colon cancer Caco-2 and HT29, liver HepG2). The cells were grown at 37 °C in a 5% CO_2_ atmosphere, with relative humidity, using culture medium DMEM/F12 (PNT2, HT29, HepG2), DMEM low glucose (DU145), DMEM high glucose (A375, HTB 140, HaCaT), MEM with NEAA (Caco-2), supplemented with 10% fetal bovine serum, and antibiotics. The cells were seeded onto 24-well plates (1.5 × 10^4^ cells/well) for 24 h before the experiment. Then, the culture medium was replaced with the same medium containing different concentrations of the tested substances from 5 to 100 µg/mL for 24 h. The LDH release was measured using the assay kit provided by Clontech, as described previously [47,48]. The supernatant from each well was transferred to a new 24-well plate briefly after the incubation with tested substances, and a proper quantity of a reagent mixture was added. After 30 min, the absorbance was measured at λ = 490 nm (the reference wavelength λ = 600 nm) using the Biotek Synergy microplate reader. Each experiment was performed in triplicate. Doxorubicin was used as a reference drug. The cytotoxicity of the samples was measured as follows: % cytotoxicity = [(Asample − Aspont): (Amax − Aspont)] × 100.

### 3.6. Statistical Analysis

Statistica v.13 was used to perform the statistical analysis (Statsoft, Tulsa, OK, USA). The obtained results were analyzed using one-way analysis of variance, followed by a post hoc Tukey’s test. All the experiments were carried out in triplicate, and the data were reported as the mean ± standard deviation. Furthermore, the differences between the groups were considered statistically significant when the *p*-values were 0.05 or less.

## 4. Conclusions

The present study proved for the first time the effect of methyl jasmonate elicitation of *Ganoderma applanatum* mycelial cultures on the production of ganoderic acid A and ganoderic acid C. The experiments showed there were differences in the accumulation of the analyzed compounds and the stimulating effects of elicitation on their production. There was no adverse effect on the biomass growth due to the applied elicitation treatments. Furthermore, the study also documented the cytotoxic effect of biomass extract obtained after the elicitation process in selected human cancer cell lines as well as the selectivity against normal cells.

Thus, *Ganoderma applanatum* mycelial cultures can be proposed as a model to study the dynamics of the accumulation of compounds with therapeutic values through abiotic elicitation.

## Figures and Tables

**Table 1:** Amounts of ganoderic acids A and C (mg/g DW ± SD) in extracts from *Ganoderma applanatum* mycelial cultures after elicitation with methyl jasmonate.

Day of Elicitor Addition in Cycle Growth of Mycelial Cultures	Elicitor Concentration MeJa	Ganoderic Acid A	Ganoderic Acid C
1	control	1.32 ± 0.03 ^ab^	0.44 ± 0.001 ^abce^
	blind trial	1.31 ± 0.02 ^ab^	0.44 ± 0.001 ^abe^
	2 µM	1.31 ± 0.12 ^ab^	0.55 ± 0.18 ^abce^
	5 µM	1.57 ± 0.12 ^ab^	0.55 ± 0.02 ^abcde^
	10 µM	1.38 ± 0.02 ^abc^	0.57 ± 0.08 ^abcde^
	50 μM	1.30 ± 0.07 ^abc^	0.45 ± 0.03 ^abcd^
	100 μM	1.45 ± 0.07 ^abc^	0.28 ± 0.01 ^abcd^
	150 μM	1.45 ± 0.08 ^bc^	0.37 ± 0.01 ^bcd^
	200 μM	1.47 ± 0.07 ^abc^	0.18 ± 0.001 ^abcde^
2	control	1.37 ± 0.14 ^abce^	0.33 ± 0.001 ^abce^
	blind trial	1.48 ± 0.01 ^abe^	0.40 ± 0.002 ^abe^
	2 µM	1.21 ± 0.11 ^abce^	0.38 ± 0.01 ^abcde^
	5 µM	1.16 ± 0.01 ^abcde^	0.45 ± 0.01 ^abcde^
	10 µM	1.23 ± 0.04 ^abcde^	0.57 ± 0.01 ^abcde^
	50 μM	1.26 ± 0.01 ^abcde^	0.88 ± 0.02 ^abcde^
	100 μM	1.09 ± 0.60 ^bcd^	0.52 ± 0.01 ^abcd^
	150 μM	1.32 ± 0.01 ^abcde^	0.72 ± 0.01 ^bcd^
	200 μM	1.56 ± 0.01 ^abe^	0.84 ± 0.02 ^abcde^
4	control	1.34 ± 0.18 ^abd^	0.42 ± 0.01 ^abce^
	blind trial	1.61 ± 0.01 ^abd^	0.54 ± 0.01 ^abe^
	2 μM	1.66 ± 0.02 ^abd^	0.57 ± 0.01 ^abcde^
	5 μM	1.34 ± 0.02 ^abcd^	0.51 ± 0.02 ^abcde^
	10 µM	1.14 ± 0.01 ^abcd^	0.61 ± 0.04 ^abcde^
	50 μM	1.38 ± 0.13 ^abcd^	0.61 ± 0.02 ^abcde^
	100 μM	1.45 ± 0.18 ^abcd^	0.63 ± 0.01 ^abcd^
	150 μM	1.51 ± 0.13 ^bc^	0.48 ± 0.01 ^bcd^
	200 μM	1.54 ± 0.05 ^abcd^	0.63 ± 0.03 ^abcde^
6	control	1.32 ± 0.01 ^a^	0.32 ± 0.001 ^a^
	blind trial	1.50 ± 0.12 ^a^	0.82 ± 0.04 ^a^
	2 µM	1.65 ± 0.08 ^a^	1.83 ± 6.85 ^a^
	5 µM	1.99 ± 0.17 ^a^	1.93 ± 0.10 ^a^
	10 µM	2.56 ± 0.12 ^a^	2.24 ± 0.05 ^a^
	50 μM	3.61 ± 1.64 ^a^	3.04 ± 0.00 ^a^
	100 μM	5.24 ± 0.43 ^a^	4.36 ± 0.06 ^a^
	150 μM	5.86 ± 0.40 ^a^	6.47 ± 1.10 ^a^
	200 μM	3.85 ± 0.06 ^a^	2.41 ± 0.13 ^a^
8	control	1.46 ± 0.03 ^a^	0.30 ± 0.001 ^abc^
	blind trial	1.55 ± 0.30 ^a^	1.68 ± 0.16 ^ac^
	2 µM	1.86 ± 0.08 ^a^	1.80 ± 0.23 ^abc^
	5 µM	2.25 ± 0.06 ^a^	1.87 ± 0.02 ^abc^
	10 µM	2.19 ± 0.03 ^a^	1.99 ± 0.31 ^abc^
	50 μM	3.10 ± 0.34 ^a^	3.53 ± 0.15 ^abc^
	100 μM	3.04 ± 0.45 ^a^	3.05 ± 0.04 ^abc^
	150 μM	3.75 ± 0.24 ^a^	3.47 ± 0.05 ^ac^
	200 μM	3.9 ± 0.22 ^a^	2.67 ± 0.62 ^abc^

The letters next to values represent Tukey’s HSD post-hoc results (*p* < 0.05 vs. control, *n* = 3).

**Table 2:** Validation of the developed methods with statistical evaluation for HPLC method.

Validation Parameters	Ganoderic Acid A	Ganoderic Acid C
t_R_ [min]	13.4	20.4
LOD [mg/mL]	0.047 S_y_ = 271,983.2056 a = 1901 × 10^4^	0.068 S_y_ = 209,786.49963 a = 1021 × 10^4^
LOQ [mg/mL]	0.143	0.205
Recovery 80% [%]	x = 100.57 S_x_ = 0.652 RSD = 0.65%	x = 99.15 S_x_ = 0.547 RSD = 0.55%
Recovery 100% [%]	x = 99.17 S_x_ = 0.610 RSD = 0.62%	x = 101.06 S_x_ = 0.656 RSD = 0.65%
Recovery 120% [%]	x = 101.02 S_x_ = 0.317 RSD = 0.31%	x = 99.18 S_x_ = 0.458 RSD = 0.46%
Precision 50% c [mg/mL]	x = 0.2588 S_x_ = 0.005227 RSD = 2.02%	x = 0.2499 S_x_ = 0.000858 RSD = 0.34%
Precision 100% c [mg/mL]	x = 0.5111 S_x_ = 0.001981 RSD = 0.39%	x = 0.5091 S_x_ = 0.001717 RSD = 0.34%
Precision 150% c [mg/mL]	x = 0.7508 S_x_ = 0.001823 RSD = 0.24%	x = 0.7502 S_x_ = 0.001152 RSD = 0.15%
Linearity	P = 1901 × 10^4^ × c + 5507 × 10^2^ r = 0.99907	P = 1021 × 10^4^ × c − 303 × 10^3^ r = 0.99808

t_R_—retention time, x—mean value, S_x_—standard deviation, RSD—relative standard deviation, c—concentration [mg/mL], S_y_—standard error of the estimate, the slope of regression line.

**Table 3:** The effect of extracts and ganoderic acid A acid on the viability of the selected cell lines expressed as IC_50_ (the concentrations that induced a 50% decrease in cell viability after 24 h of treatment).

	IC_50_ [μg/mL]		
	Skin cell line panel		
	A375	HTB140	HaCaT
E1	16.6	nd	45.9
E2	49.6	nd	nd
Ganoderic acid A	87.1	nd	50.7
Doxorubicin	0.59	5.71	4.68
	Prostate cell line panel		
	DU145	PC3	PNT2
E1	12.7	38.4	nd
E2	25.4	46.8	nd
Ganoderic acid A	79.3	nd	nd
Doxorubicin	3.18	>50	1.38
	Gastrointestinal tract cell lines panel		
	Caco-2	HT29	HepG2
E1	25.2	nd	nd
E2	9.93	59.8	nd
Ganoderic acid A	63.4	42.9	nd
Doxorubicin	3.44	1.53	nd

E1—control extract; E2—extract obtained after elicitation (variant corresponding to the highest concentration of triterpenes), nd—not detected.

## Data Availability

Not applicable.

## Supplementary Materials

The following supporting information can be downloaded at: https://www.mdpi.com/article/10.3390/plants12020294/s1, Table S1: Statistical differences for cytotoxic activity of *Ganoderma applanatum* extracts and ganoderic acid A; (5–100 µg/mL) to prostate cell line panel—DU145, PC3, PNT2; Table S2: Statistical differences for cytotoxic activity of *Ganoderma applanatum* extracts and ganoderic acid A; (5–100 µg/mL) to skin cell line panel—A375, HTB140, HaCaT; Table S3: Statistical differences for cytotoxic activity of *Ganoderma applanatum* extracts and ganoderic acid A; (5–100 µg/mL) to gastrointestinal tract cell lines panel–Caco-2, HT29, HepG2.

## Sample Availability

Samples of the compounds are available from the authors.
