# Supplementary material for: Effect of Methyl Jasmonate Elicitation on Triterpene Production and Evaluation of Cytotoxic Activity of Mycelial Culture Extracts of Ganoderma applanatum (Pers.) Pat
Source: Plants (Basel). 2023 Jan 8;12(2):294. doi: 10.3390/plants12020294 (PMC9867392; doi:10.3390/plants12020294)
Supplement: Supplementary file 1 [file plants-12-00294-s001.zip › Tabele S2.pdf]

Table S2. Statistical differences for cytotoxic activity of *Ganoderma applanatum* extracts and ganoderic acid A; (5-100 µg/mL) to skin cell line panel – A375, HTB140, HaCaT

**E1– control extract A375**	5 µg/mL	10 µg/mL	20 µg/mL	30 µg/mL	40 µg/mL	50 µg/mL	100 µg/mL
5 µg/mL							
10 µg/mL							
20 µg/mL							
30 µg/mL							
40 µg/mL							
50 µg/mL							
100 µg/mL							
	Not significant		P<0.05		P<0.01		P<0.001

**E2 – extract after elicitation A375**	5 µg/mL	10 µg/mL	20 µg/mL	30 µg/mL	40 µg/mL	50 µg/mL	100 µg/mL
5 µg/mL							
10 µg/mL							
20 µg/mL							
30 µg/mL							
40 µg/mL							
50 µg/mL							
100 µg/mL							
	Not significant		P<0.05		P<0.01		P<0.001

**Ganoderic acid A A375**	5 µg/mL	10 µg/mL	20 µg/mL	30 µg/mL	40 µg/mL	50 µg/mL	100 µg/mL
5 µg/mL							
10 µg/mL							

20 µg/mL							
30 µg/mL							
40 µg/mL							
50 µg/mL							
100 µg/mL							
	Not significant		P<0.05		P<0.01		P<0.001

**E1– control extract HTB140**	5 µg/mL	10 µg/mL	20 µg/mL	30 µg/mL	40 µg/mL	50 µg/mL	100 µg/mL
5 µg/mL							
10 µg/mL							
20 µg/mL							
30 µg/mL							
40 µg/mL							
50 µg/mL							
100 µg/mL							
	Not significant		P<0.05		P<0.01		P<0.001

**E2 – extract after elicitation HTB140**	5 µg/mL	10 µg/mL	20 µg/mL	30 µg/mL	40 µg/mL	50 µg/mL	100 µg/mL
5 µg/mL							
10 µg/mL							
20 µg/mL							
30 µg/mL							
40 µg/mL							
50 µg/mL							
100 µg/mL							
	Not significant		P<0.05		P<0.01		P<0.001

**Ganoderic acid A HTB140**	5 µg/mL	10 µg/mL	20 µg/mL	30 µg/mL	40 µg/mL	50 µg/mL	100 µg/mL
5 µg/mL							
10 µg/mL							
20 µg/mL							
30 µg/mL							
40 µg/mL							
50 µg/mL							
100 µg/mL							
	Not significant		P<0.05		P<0.01		P<0.001

**E1– control extract HaCaT**	5 µg/mL	10 µg/mL	20 µg/mL	30 µg/mL	40 µg/mL	50 µg/mL	100 µg/mL
5 µg/mL							
10 µg/mL							
20 µg/mL							
30 µg/mL							
40 µg/mL							
50 µg/mL							
100 µg/mL							
	Not significant		P<0.05		P<0.01		P<0.001

**E2 – extract after elicitation HaCaT**	5 µg/mL	10 µg/mL	20 µg/mL	30 µg/mL	40 µg/mL	50 µg/mL	100 µg/mL
5 µg/mL							
10 µg/mL							

20 μg/mL							
30 μg/mL							
40 μg/mL							
50 μg/mL							
100 μg/mL							
	Not significant		P<0.05		P<0.01		P<0.001

**Ganoderic acid A HaCaT**	5 μg/mL	10 μg/mL	20 μg/mL	30 μg/mL	40 μg/mL	50 μg/mL	100 μg/mL
5 μg/mL							
10 μg/mL							
20 μg/mL							
30 μg/mL							
40 μg/mL							
50 μg/mL							
100 μg/mL							
	Not significant		P<0.05		P<0.01		P<0.001
