# Supplementary material for: Effect of Methyl Jasmonate Elicitation on Triterpene Production and Evaluation of Cytotoxic Activity of Mycelial Culture Extracts of Ganoderma applanatum (Pers.) Pat
Source: Plants (Basel). 2023 Jan 8;12(2):294. doi: 10.3390/plants12020294 (PMC9867392; doi:10.3390/plants12020294)
Supplement: Supplementary file 1 [file plants-12-00294-s001.zip › Tabele S3.pdf]

Table S3. Statistical differences for cytotoxic activity of *Ganoderma applanatum* extracts and ganoderic acid A; (5-100 µg/mL) to gastrointestinal tract cell lines panel – Caco-2, HT29, HepG2

**E1– control extract Caco-2**	5 µg/mL	10 µg/mL	20 µg/mL	30 µg/mL	40 µg/mL	50 µg/mL	100 µg/mL
5 µg/mL							
10 µg/mL							
20 µg/mL							
30 µg/mL							
40 µg/mL							
50 µg/mL							
100 µg/mL							
	Not significant		P<0.05		P<0.01		P<0.001

**E2 – extract after elicitation Caco-2**	5 µg/mL	10 µg/mL	20 µg/mL	30 µg/mL	40 µg/mL	50 µg/mL	100 µg/mL
5 µg/mL							
10 µg/mL							
20 µg/mL							
30 µg/mL							
40 µg/mL							
50 µg/mL							
100 µg/mL							
	Not significant		P<0.05		P<0.01		P<0.001

**Ganoderic acid A Caco-2**	5 µg/mL	10 µg/mL	20 µg/mL	30 µg/mL	40 µg/mL	50 µg/mL	100 µg/mL
5 µg/mL							

10 µg/mL							
20 µg/mL							
30 µg/mL							
40 µg/mL							
50 µg/mL							
100 µg/mL							
	Not significant		P<0.05		P<0.01		P<0.001

**E1– control extract HT-29**	5 µg/mL	10 µg/mL	20 µg/mL	30 µg/mL	40 µg/mL	50 µg/mL	100 µg/mL
5 µg/mL							
10 µg/mL							
20 µg/mL							
30 µg/mL							
40 µg/mL							
50 µg/mL							
100 µg/mL							
	Not significant		P<0.05		P<0.01		P<0.001

**E2 – extract after elicitation HT-29**	5 µg/mL	10 µg/mL	20 µg/mL	30 µg/mL	40 µg/mL	50 µg/mL	100 µg/mL
5 µg/mL							
10 µg/mL							
20 µg/mL							
30 µg/mL							
40 µg/mL							
50 µg/mL							
100 µg/mL							

	Not significant		P<0.05		P<0.01		P<0.001

**Ganoderic acid A HT-29**	5 µg/mL	10 µg/mL	20 µg/mL	30 µg/mL	40 µg/mL	50 µg/mL	100 µg/mL
5 µg/mL							
10 µg/mL							
20 µg/mL							
30 µg/mL							
40 µg/mL							
50 µg/mL							
100 µg/mL							
	Not significant		P<0.05		P<0.01		P<0.001

**E1– control extract HepG2**	5 µg/mL	10 µg/mL	20 µg/mL	30 µg/mL	40 µg/mL	50 µg/mL	100 µg/mL
5 µg/mL							
10 µg/mL							
20 µg/mL							
30 µg/mL							
40 µg/mL							
50 µg/mL							
100 µg/mL							
	Not significant		P<0.05		P<0.01		P<0.001

**E2 – extract after elicitation HepG2**	5 µg/mL	10 µg/mL	20 µg/mL	30 µg/mL	40 µg/mL	50 µg/mL	100 µg/mL
5 µg/mL							

10 µg/mL							
20 µg/mL							
30 µg/mL							
40 µg/mL							
50 µg/mL							
100 µg/mL							
	Not significant		P<0.05		P<0.01		P<0.001

**Ganoderic acid A HepG2**	5 µg/mL	10 µg/mL	20 µg/mL	30 µg/mL	40 µg/mL	50 µg/mL	100 µg/mL
5 µg/mL							
10 µg/mL							
20 µg/mL							
30 µg/mL							
40 µg/mL							
50 µg/mL							
100 µg/mL							
	Not significant		P<0.05		P<0.01		P<0.001
